# Meta-analysis of liver injury in patients with COVID-19

**DOI:** 10.1097/MD.0000000000034320

**Published:** 2023-07-21

**Authors:** Xinghai Li, Caiping Fan, Jin Tang, Ning Zhang

**Affiliations:** a Department of Minimally Invasive Intervention, Ganzhou People’s Hospital, Ganzhou, China; b Department of Gastroenterology, Ganzhou People’s Hospital, Ganzhou, China.

**Keywords:** COVID-19, incidence, liver injury, meta-analysis, risk factors

## Abstract

The coronavirus disease 2019 (COVID-19) is a major public health problem threatening human health. It can lead to multiple system complications, among which liver damage is also a common complication of COVID-19. The pathogenesis of liver injury is complex and involves the interaction of multiple factors. This study aims to investigate the incidence and risk factors of liver injury in COVID-19 patients and analyze the impact of liver injury on clinical prognosis of patients, so as to provide corresponding basis for clinical diagnosis and treatment.

**Methods:**

PubMed and Cochrane Library were searched in computer to collect original studies on liver injury cases, laboratory indicators and clinical outcomes in COVID-19 patients. Articles were screened according to inclusion and exclusion criteria, and data were meta-analyzed using Stata12.0 software.

**Results:**

A total of 49 studies, including 23,611 patients with COVID-19, had a prevalence of liver injury of 39.63%. Subgroup analysis found that patients in the Americas had the highest incidence of liver injury at 43.7% and lowest in Africa (25.99%). The vast majority of liver injury is manifested by aminotransferase or bilirubin levels greater than 1 times the upper limit of normal (49.16%). The older the age, the male, the associated chronic liver disease, and the higher the levels of white blood cells, neutrophils, and C-reactive protein, the higher the risk of liver injury. The use of hormones, hydroxychloroquine, and tocilizumab increases the risk of liver injury. Patients with concurrent liver injury have longer hospital stays, are more likely to progress to severe cases, and have a higher risk of death than patients without liver injury.

**Conclusion:**

The incidence of liver injury in COVID-19 patients was high, affected by age, gender, chronic liver disease, inflammatory state and medication, and patients with liver injury were hospitalized longer and were more likely to have a poor prognosis. Therefore, clinical attention should be paid to early intervention.

## 1. Introduction

Since December 2019, the severe acute respiratory syndrome coronavirus-2 (SARS-CoV-2) infection has become the world’s largest public health problem, and there is no specific vaccine or targeted drug treatment to date. Coronavirus disease 2019 (COVID-19) mainly targets the respiratory system, and common clinical manifestations of infected patients are fever, cough, and dyspnea. Some studies have shown that the virus can also damage the heart, kidneys, digestion and nervous system, so patients can also have many extrapulmonary manifestations, such as diarrhea, vomiting and abdominal pain and other gastrointestinal symptoms, severe cases can even appear sepsis, acute myocardial injury, multiple organ failure and a series of serious complications.^[1,2]^ In 2019, scholars reported that 43 of the 99 COVID-19 patients in Wuhan had liver injury.^[3]^ Since then, many studies have found that patients with COVID-19 are at risk of liver injury. The pathogenesis of liver injury is complex, involving multiple links and multiple factors affecting each other. The mechanism of liver injury currently discussed include direct virus-induced liver injury, immune stress-mediated liver injury, tissue hypoxia secondary to multi-organ dysfunction, and drug-induced liver injury. Abnormal markers of liver injury are characterized by elevated levels of alanine aminotransferase (ALT), aspartate aminotransferase (AST), γ-glutamyl transferase, and bilirubin, which lead to functional impairment of hepatocytes and eventually to cholangiocyte dysfunction. The incidence of liver injury reported by each study varies widely.^[4–6]^ This study aims to systematically analyze the published relevant studies to explore the incidence and risk factors of liver injury in COVID-19 patients, and analyze the impact of liver injury on the clinical prognosis of patients, so as to provide corresponding basis for clinical diagnosis and treatment.

## 2. Information and methods

### 2.1. Literature search strategy

The studies on liver injury and COVID-19 published in PubMed and Cochrane Library were searched by computer. Keywords include COVID-19, 2019-nCoV, SARS-CoV-2, liver injury, liver damage, hepatic injury, abnormal liver function. The search was conducted in the above database with OR or AND as the conjunctive. The search time was from December 2019 to January 31, 2023.

### 2.2. Eligibility criteria

To be eligible for this meta-analysis, studies must meet the following inclusion criteria: observational study; studies reports the incidence of liver injury, laboratory indicators, or clinical outcomes among patients diagnosed with COVID-19. Repeated published studies, studies with incomplete data, animal experiments, case reports, meta-analyses were excluded. Two review authors searched the above literature databases separately and screened them according to the inclusion and exclusion criteria.

### 2.3. Evaluation criteria for liver injury

Definitions of liver injury varied across studies, with some studies defining liver injury with any aminotransferase or bilirubin level greater than 1 times the upper limit of normal. Some studies define any aminotransferase level greater than 2 times the upper limit of normal (ULN) as liver injury. Some studies have also defined ALT elevation ≥ 5 times ULN alone, or alkaline phosphatase (ALP) alone ≥2 times ULN, as liver injury.

### 2.4. Literature quality evaluation

The quality evaluation of the included literature referred to the Newcastle Ottawa Scale scoring standard, including 3 categories of selection bias, comparability, and exposure, a total of 8 scoring items, and the higher the evaluation score, the better the quality of the literature.

### 2.5. Statistical analysis

This study performed meta-analysis of all data using the Stata12.0 software (StataCorp LP 4905Lakeway Drive College Station, USA). The *Q* test was used to evaluate the heterogeneity between the studies, and when the *P* > .1, *I*^2^ ≤ 50%, the included studies were considered to have no significant heterogeneity, and the fixed-effect model was selected for pooling. Instead, a random effect model is used. Continuous variables are represented by standardized mean differences and their 95% confidence interval (CI), and dichotomous variables were represented by odds ratio (OR) and their 95% CI, and the difference was considered statistically significant when *P* < .05.

## 3. Results

### 3.1. Literature search results

**Figure 1.**
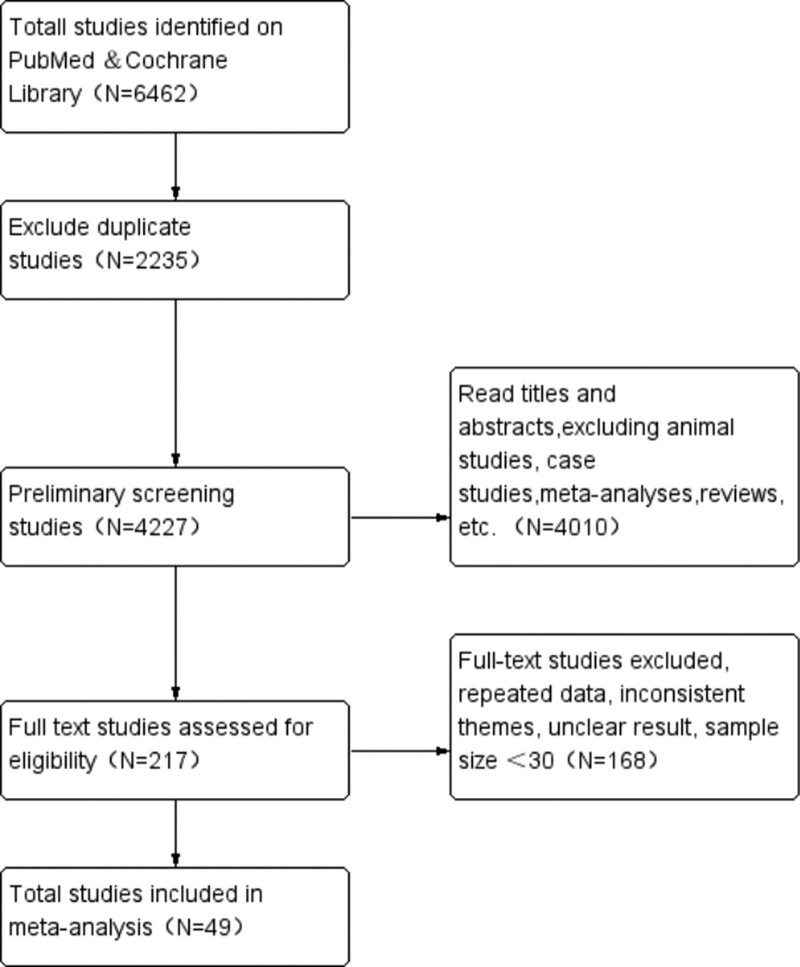
Flow chart of the included studies.

At the initial examination, 6462 studies were obtained, which were screened and evaluated layer by layer according to inclusion and exclusion criteria, and 49 studies were finally included, most of which were retrospective studies, from 10 countries, with a total of 23,611 COVID-19 patients, including 9357 cases complicated with liver injury.^[7–55]^ The flow chart of literature screening is shown in Figure 1. The basic characteristics of the included literature are shown in Table 1.

**Table 1:** The characteristics and quality of studies included in the meta-analyze

Study	Research type	Country/area	Diagnostic criteria for liver injury (ALT, AST or TBIL)	Cases	Mean age (SD, yr)	Liver injury	Prognostic indicator	Lisk factor	Quality score
1. Hemamala 2021^[7]^	Retrospective	India	>1 ULN	445	49.6 (2.2)	298	ab	①②③	7
2. Wang 2020^[8]^	Retrospective	China/Beijing	>1 ULN	105	45	59	a	NR	7
3. Bogline 2022^[9]^	Retrospective	Italy	>1 ULN	434	72	123	NR	②	7
4. Jiang 2020^[10]^	Retrospective	China/Zhejiang	>1 ULN	131	51.2 (16.1)	76	ac	NR	7
5. Shen 2021^[11]^	Retrospective	China/Hubei	>1 ULN	356	52.11 (17.9)	177	ac	①②③④⑤⑥	8
6. Desai 2020^[12]^	Retrospective	America	>1 ULN	639	58.89 (15.61)	476	b	①②	8
7. Shousha 2021^[13]^	Cohort study	Egypt	>1 ULN	428	45.04(17.61)	137	b	②⑦	8
8. Li 2020^[14]^	Retrospective	China/Anhui	>1 ULN	159	43	28	a	NR	6
9. Kalal 2021^[15]^	Retrospective	India	>1 ULN	184	45.79 (13.48)	95	abc	①②	7
10. Wang 2020^[16]^	Cohort study	China/Wuhan	>1 ULN	657	62.89 (3.38)	303	a	①②③④⑤⑦	8
11. Yang 2020^[17]^	Retrospective	China/Wuhan	>1 ULN	136	54.59 (14.99)	18	a	NR	6
12. Guo 2020^[18]^	Retrospective	China/Shanghai	>1 ULN	332	50 (20.85)	98	a	①②⑦	8
13. Qu 2021^[19]^	Retrospective	China/Hunan	>1 ULN	266	NR	38	NR	③④⑤	6
14. Wang 2020^[20]^	Cohort study	China/Wuhan	>1 ULN	339	71 (8)	96	b	NR	7
15. Qi 2020^[21]^	Cohort study	China	>1 ULN	70	NR	32	c	①②③④⑤	7
16. Piano 2020^[22]^	Retrospective	Italy	>1 ULN	565	66 (15)	329	bc	①②③④⑤⑥⑦⑧	7
17. Phipps 2020^[23]^	Retrospective	America	>2 ULN	2273	64.99 (3.47)	489	ab	②⑦	7
18. Mishra 2021^[24]^	Retrospective	America	>1 ULN	348	61.5 (15.2)	184	c	①②③④⑤⑥⑦⑧	8
19. Fan 2020^[25]^	Retrospective	China/Shanghai	>1 ULN	148	50 (5.31)	55	abc	①②③④⑥⑦⑧	8
20. Chew 2021^[26]^	Cohort study	America	>5 ULN	834	67.84 (13.07)	105	bc	①②⑦⑧	8
21. Chen 2021^[27]^	Cohort study	China/Wuhan	>1 ULN	830	51 (30)	227	ab	①②④⑥⑦	7
22. Yang 2020^[28]^	Retrospective	China/Wuhan	Undefine	52	59.7 (13.3)	15	b	NR	6
23. Krishnan 2022^[29]^	Retrospective	America	>1 ULN	3830	63.67 (20.54)	2698	ab	NR	7
24. Zhang 2021^[30]^	Retrospective	China/Wuhan	>1 ULN	440	63 (25)	254	abc	①②⑦	7
25. BJ 2021^[31]^	Retrospective	Netherlands	>1 ULN	382	68	159	abc	②	8
26. Chu 2020^[32]^	Cohort study	China/Wuhan	>2 ULN	838	NR	429	ab	①②③④⑤⑥	7
27. Hassanin 2021^[33]^	Retrospective	Egypt	>2 ULN	1238	54	296	c	①②③④	7
28. Xie 2020^[34]^	Retrospective	China/Wuhan	>1 ULN	79	59.61 (12.42)	29	c	①②③④⑤⑥	8
29. Wan 2020^[35]^	Retrospective	China/Chongqing	>1 ULN	135	45.95 (14.24)	30	a	NR	6
30. Cai 2020^[36]^	Cohort study	China/Shenzhen	>1 ULN	417	47 (19.34)	192	a	②⑦	8
31. Ma 2021^[37]^	Retrospective	China/Shanghai	>1 ULN	109	62.79 (4.25)	39	a	②	7
32. Zhang 2020^[38]^	Retrospective	China/Wuhan	>1 ULN	218	50.1 (18.4)	79	a	①②③④⑤⑥⑦	7
33. Wang 2021^[39]^	Retrospective	China/Wuhan	>1 ULN	211	54 (4.72)	80	a	NR	8
34. Xu 2020^[40]^	Retrospective	China/Wuhan	>1 ULN	62	41	10	a	NR	6
35. Fu 2020^[41]^	Retrospective	China/Wuhan	>1 ULN	482	56.35 (12.78)	142	abc	①②③④⑥⑦⑧	8
36. Wang 2020^[42]^	Retrospective	China/Wuhan	>1 ULN	69	46.59 (20.44)	24	a	NR	7
37. Huang 2020^[43]^	Prospective	China/Wuhan	>1 ULN	41	49.35 (13.06)	15	a	NR	6
38. Zhou 2020^[44]^	Retrospective	China/Wuhan	>1 ULN	189	56.35 (15.69)	59	b	NR	8
39. Cao 2020^[45]^	Retrospective	China/Wuhan	Undefine	102	51.54 (20.3)	34	b	NR	7
40. Zhang 2020^[46]^	Retrospective	China/Wuhan	>1 ULN	40	46.38 (3.11)	16	ab	①②③④⑤⑥⑧	7
41. Zhao 2020^[47]^	Retrospective	China/Jingzhou	>1 ULN	91	46	18	a	NR	6
42. Kumar 2020^[48]^	Cross-sectional	India	>1 ULN	91	44.54 (13.59)	70	NR	①②	7
43. Sadeghi 2020^[49]^	Retrospective	Iran	>1 ULN	102	55.13 (17.02)	65	c	①②③④⑤⑥	8
44. Ben 2020^[50]^	Retrospective	America	>4 ULN	176	NR	109	bc	①②⑥⑧	8
45. Mihai 2022^[51]^	Retrospective	Canada	>1 ULN	249	NR	157	NR	③④⑤⑥	8
46. Siddiqui 2021^[52]^	Retrospective	America	>4 ULN	1935	NR	396	NR	①②⑥⑦	7
47. Faghih 2022^[53]^	Cohort study	Iran	>1 ULN	1017	62.58 (17.45)	324	ab	②	6
48. Garrido 2021^[54]^	Retrospective	Portugal	>1 ULN	291	NR	122	abc	①②⑦	7
49. Cholongitas 2022^[55]^	Retrospective	Greece	>5 ULN	1046	63.5 (17)	53	c	①②③⑥⑧	7

a = non-severe VS severe, b =sSurvival VS death, c = hospital stays, ALT = alanine aminotransferase, AST = aspartate transaminase, NR = Not repord, TBIL = total bilirubin abnormal, ULN = upper limit of normal.

① = age, ② = gender, ③ = leukocyte level, ④ = lymphocyte count, ⑤ = neutrophil count, ⑥ = C-reactive protein, ⑦ = chronic liver disease, ⑧ = medicine.

### 3.2. Incidence of liver injury in patients with COVID-19

**Figure 2.**
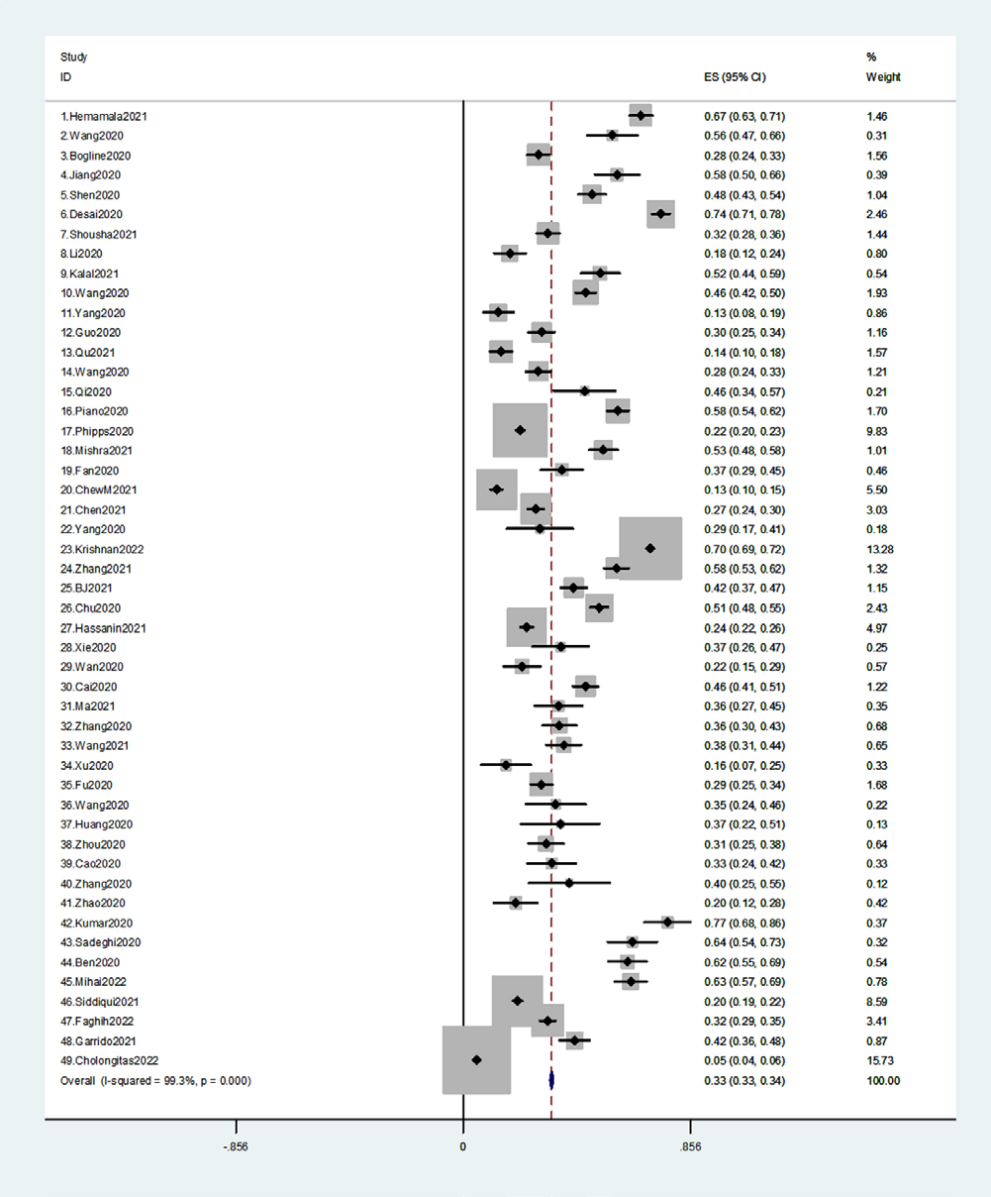
The incidence of liver injury in patients with COVID-19.

The 49 included studies all reported the incidence of liver injury in patients with COVID-19, with a total of 23,611 participants, and the incidence of liver injury reported in each study ranged from 5.07% to 76.92%. The overall incidence of liver injury in COVID-19 patients was 39.63% (9357/23,611), standard error = 0.33, 95% CI: 0.32–0.34, *P* = .0001. (The results are shown in Fig. 2, Table 2.)

**Table 2:** Subgroup analysis of the incidence of liver injury in patients with COVID-19

Subgroup	Study	Cases	The overall incidence of liver injury %	*I*^2^ (%)	*P*_1_	ES (95%CI)	*P*_2_
Total	49	23,611	39.63	99.3	.0001	0.33 (0.32, 0.34)	.0001
Subgroups of countries							
Asian	33	7926	40.37	96.4	.0001	0.38 (0.37, 0.39)	.0001
America	9	11,301	43.7	99.8	.0001	0.40 (0.39, 0.41)	.0001
Europe	5	2718	28.92	99.5	.0001	0.15 (0.13, 0.16)	.0001
Africa	2	1666	25.99	90.1	.002	0.26 (0.24, 0.28)	.0001
Subgroup defined by liver injury							
>1 ULN	40	15,117	49.16	98.6	.0001	0.48 (0.47, 0.49)	.0001
>2 ULN	3	4349	27.91	99.2	.0001	0.26 (0.25, 0.28)	.0001
>3 ULN	2	2111	23.92	99.2	.0001	0.23 (0.21, 0.25)	.0001
>5 ULN	2	1880	8.40	96.8	.0001	0.07 (0.06, 0.08)	.0001
Undefind	2	154	31.82	0	.568	0.32 (0.24, 0.39)	.0001

COVID-19 = coronavirus disease 2019, ES = standard error.

Subgroup analysis by country found that COVID-19 patients from the Americas had the highest incidence of liver injury at 43.7%, Asian patients with 40.37%, European patients with 28.92%, and Africa had the lowest incidence of liver injury at 25.99%. (The results are shown in Table 2.)

Subgroup analysis based on the definition of liver injury found that 40 studies defined any aminotransferase or bilirubin level greater than 1 times the upper limit of normal as liver injury (>1 ULN group), with a total of 15,117 patients with CVOVID-19, who had the highest incidence of liver injury in this group at 49.16%. Three studies in the >2 ULN group had a prevalence of liver injury of 27.91%. Two studies in the >3 ULN group had a prevalence of liver injury of 23.92%. Two studies had the lowest incidence of liver injury in the >5 ULN group at 8.4%. Two other studies did not have a clear definition of liver injury, with a total of 154 patients with COVID-19 and a prevalence of liver injury of 31.82%. (The results are shown in Table 2.)

### 3.3. Risk factors for liver injury in patients with COVID-19

#### 3.3.1. Association between liver injury and clinical features in patients with COVID-19.

Twenty-five studies compared the age of COVID-19 patients with liver injury to those without liver injury, and 33 studies compared the gender of patients in both groups. The results of meta-analysis showed that there were statistical differences in age and gender between the 2 groups, and the mean age and male proportion in the liver injury group were higher than those in the non-liver injury group. (The results are shown in Table 3.)

**Table 3:** Meta-analysis of risk factors and clinical prognosis associated with liver injury in patients with COVID-19.

Research contents	Study	*I*^2^ (%)	Effect model	OR/SMD (95% CI)	*P*	Publication bias	
						Egger (*P*)	Begg (*P*)
Age	25	98.5	RE	0.51 (0.15, 0.87)	.005	0.79 (.439)	2.64 (.008)
Gender	33	81.5	RE	1.89 (1.58, 2.25)	.0001	0.27 (.788)	0.39 (.698)
Leukocyte level	17	99	RE	0.86 (0.23, 1.84)	.007	0.54 (.598)	1.44 (.149)
Lymphocyte count	16	97.7	RE	−0.30 (−0.66, −0.07)	.109	−1.32 (.210)	0.05 (.964)
Neutrophil count	12	89.2	RE	0.63 (0.41, 0.85)	.0001	0.60 (.562)	0.48 (.631)
C-reactive protein	15	96.5	RE	0.83 (0.77, 0.89)	.0001	1.63 (.127)	1.48 (.138)
Chronic liver disease	15	17.6	FE	1.52 (1.25, 1.85)	.0001	1.35 (.201)	0.69 (.488)
Antibiotic	7	80.5	RE	1.59 (0.98, 2.58)	.062	2.64 (.047)	1.8 (.072)
Antiviral	5	68.7	RE	0.84 (0.47, 1.49)	.547	1.20 (.297)	1.13 (.260)
Glucocorticoid	6	83	RE	1.90 (1.04, 3.45)	.037	0.39 (.717)	0.00 (1.00)
Hydroxychloroquine	4	0	FE	2.00 (1.34, 2.98)	.001	4.86 (.04)	1.70 (.089)
Tocilizumab	5	46	FE	3.58 (2.43, 5.27)	.0001	−0.13 (.903)	−0.24 (1.00)
Non-severe vs severe	29	77.5	RE	0.40 (0.32, 0.49)	.0001	−0.03 (.975)	0.84 (.399)
Hospital stays	15	99.4	RE	1.13 (0.20, 2.06)	.0001	1.35 (.199)	1.78 (.075)
Survival vs death	22	77.8	RE	0.44 (0.34, 0.57)	.0001	−2.79 (.011)	1.58 (.114)

COVID-19 = coronavirus disease 2019, FE = fixed-effect model, OR = odds ratio, RE = random effect model, SMD = standardized mean differences.

#### 3.3.2. Association between laboratory markers and liver injury in patients with COVID-19.

Seventeen studies compared Leukocyte level in the liver injury group with non-liver injury in people with CIOVID-19, 16 studies compared lymphocyte counts in both groups, 12 studies compared neutrophil counts, and 15 studies compared C-reactive protein levels. The results of meta-analysis showed that there were statistically significant differences in Leukocyte level, neutrophil count and C-reactive protein levels in the 2 groups, among which the leukocyte level, neutrophil count and C-reactive protein levels in the liver injury group were higher than those in the non-liver injury group. However, there was no statistically significant difference in lymphocyte counts between the 2 groups. (The results are shown in Table 3.)

#### 3.3.3. Association between liver injury and chronic liver disease in patients with COVID-19.

Fifteen studies reported chronic liver disease, with small heterogeneity between studies (*I*^2^ = 17.6%), and the results showed that the proportion of chronic liver disease in the liver injury group was higher than that in the non-liver injury group. (The results are shown in Table 3.)

#### 3.3.4. Association of liver injury and pre-admission pharmacotherapy in patients with COVID-19.

Eight studies reported on pharmacological treatments for people with COVID-19 prior to admission, including antibiotics, antivirals, glucocorticoids, hydroxychloroquine, and tocilizumab. Among them, there was no significant difference in the use of antibiotics and antivirals between the liver injury group and the non-liver injury group. There were statistical differences in the use of glucocorticoids, hydroxychloroquine and tocilizumab between the 2 groups, and the proportion of the liver injury group using the above 3 drugs was higher than that of the non-liver injury group. (The results are shown in Table 3.)

### 3.4. Association between liver injury and clinical prognosis in patients with COVID-19

#### 3.4.1. Association between liver injury and disease severity.

Twenty-nine studies reported the number of patients with liver injury in the severe group and non-severe group, with large heterogeneity between studies (*I*^2^ = 77.5%), and the meta-analysis result showed that the proportion of liver injury in the severe group was higher than that in the non-severe group (OR = 0.40, 95% CI: 0.32–0.49, *P* = .0001), suggesting that there was an association between liver injury complicated by COVID-19 patients and progression to severe cases. (The results are shown in Table 3.)

#### 3.4.2. Association between liver injury and length of hospital stay.

Fifteen studies compared the length of hospital stay between the 2 groups, with significant heterogeneity between studies (*I*^2^ = 99.4%), and the use of random effect model pooling suggested that COVID-19 patients with liver injury had significantly longer hospital stay than that of normal groups (standardized mean differences = 0.70, 95% CI: 0.63–0.77, *P* = .0001). (The results are shown in Table 3.)

#### 3.4.3. Association between liver injury and patient mortality.

Twenty-two studies reported the number of patients with liver injury in the survival group and the death group, with large heterogeneity between studies (*I*^2^ = 77.8%), and the results showed that the proportion of patients with COVID-19 complicated by liver injury was higher than that in the survival group (OR = 0.44, 95% CI: 0.34–0.57, *P* = .0001), indicating that the risk of death after complicated liver injury in COVID-19 patients was significantly increased. (The results are shown in Table 3.)

### 3.5. Sensitivity analyses and publication bias

Sensitivity analyses of major risk factors (including age, gender, Leukocyte level, lymphocyte count, neutrophil count, C-reactive protein, chronic liver disease), drug therapy (including antibiotics, antivirals, glucocorticoids, hydroxychloroquine, tocilizumab), and clinical prognostic indicators failed to identify sources of heterogeneity. Egger’s and Begg’s tests were used to assess publication bias. Based on the results of Egger’s and Begg’s tests, there was no significant publication bias. (The results are shown in Table 3.)

## 4. Discussion

Unlike previous studies that defined liver injury as elevated ALT greater than 5 times the upper limit of normal and/or ALP greater than 2 times the upper limit of normal, COVID-19-related liver injury is defined as any liver injury that occurs during disease progression and COVID-19 treatment in patients with or without preexisting liver disease.^[56,57]^ The main manifestations were elevated serum liver biochemical indexes, mainly AST and ALT, and bilirubin.^[57]^ Depending on the pattern of liver injury, it can be further classified as hepatocellular liver injury, cholestatic liver injury, and mixed liver injury. The pathogenesis of liver injury is complex, and the proposed mechanisms mainly include direct induction by virus, storm of inflammatory factors, tissue hypoxia secondary to multi-organ dysfunction, drug-induced liver injury, and reactivation of previous liver disease.^[58]^

SARS-CoV-2 is a β coronavirus that enters human cells by binding to angiotensin-converting enzyme 2 (ACE2) membrane receptors on the surface of human cells, participating in the body’s immune regulation, which in turn triggers the inflammatory response. ACE2 membrane receptors are widely distributed in the lungs, heart, kidneys, gastrointestinal tract, and brain. Studies have found that ACE2 is highly expressed in the liver, especially in the intrahepatic bile duct epithelial cells.^[59]^ Therefore, the liver is also one of the target organs for COVID-19 attack. Deng et al^[60]^ compared the clinical characteristics of liver injury in patients infected with Delta and Omicron variants, and found that the liver injury secondary to the 2 variants was mainly caused by elevated cholangiocyte injury parameters (TBA, γ-glutamyl transferase or ALP), and there was no significant difference in the incidence of liver injury between the 2 types. The study suggests that infection with Delta and Omicron viruses directly induces cholangiocyte damage because the receptor for COVID-19 (ACE 2) is expressed more in cholangiocyte than in hepatocytes.^[60]^ Moderate microvascular steatosis and slight lobular activity have also been observed in liver biopsies of patients with COVIFD-19, often presenting clinically as acute hepatitis with elevated AST, ALT, and total bilirubin levels.^[61,62]^

The incidence of liver injury reported by studies varied widely. This study included 49 articles for analysis, and the total incidence of liver injury was 39.63%. Subgroup analysis found that the rate of liver injury varied widely by region, In order to evaluate the source of this difference, we conducted subgroup analysis based on different definitions of liver injury in various studies, and found that most of the liver injury may be mild liver injury with transaminase or bilirubin levels greater than 1 times the upper limit of normal. In this study, several common laboratory results and predisposing factors were statistically analyzed, and 10 indicators were found to be associated with liver injury in patients with COVID-19.

COVID-19-related liver injury was significantly correlated with gender and age. Male patients are more likely to develop liver injury than women, which may be related to higher levels of androgens in men, lack of estrogen’s protective effect on the liver, and more ACE2 receptors expressed in the body.^[63]^ A large cohort study found that patients aged 40 to 69 years were at particularly high risk of liver injury and liver-related death,^[64]^ this study also found that the average age of patients with liver injury was higher than that of the control group, suggesting that advanced age is also one of the risk factors for liver injury. This may be due to the fact that older people often have more underlying medical conditions and that their immunity is worse than that of younger people. Therefore, more attention should be paid to the changes of liver function in middle-aged and elderly male patients.

Severe COVID-19 can cause systemic inflammatory responses that cause cytokine storms and lead to multiple organ failure. And in this process, studies have found a positive correlation between serum inflammatory cytokine levels and markers of liver dysfunction in COVID-19 patients, suggesting that systemic inflammatory responses and cytokine storms are also involved in liver injury.^[65]^ This study found that the levels of white blood cells, neutrophils and C-reactive protein in patients with liver injury were higher than those without liver injury. The results suggest that these inflammatory factors are associated with the development of liver injury and may be risk factors for the development of liver injury.

In addition, ischemic hypoxic hepatitis is also one of the liver injuries that cannot be ignored in patients with severe COVID-19. Hypoxia due to COVID-19-related complications such as respiratory distress syndrome or multi-organ failure may also lead to hepatic ischemia and hypoxic perfusion dysfunction, further inducing liver injury.^[57]^

Previous studies have suggested no clear correlation between chronic liver disease and disease severity in patients with COVID-19.^[66,67]^ This study found that the proportion of patients with liver injury complicated with chronic liver disease was higher than that in patients with non-liver injury, which suggested that chronic liver disease was one of the risk factors for liver injury. This may be due to the fact that patients with chronic liver disease have a long-term state of inflammatory stress on the liver, and ACE 2 expression may be higher than in patients without liver disease.^[57]^

Drug-induced liver injury has also received widespread attention. Cai et al^[36]^ analyzed the liver function indicators of 417 COVID-19 patients in Shenzhen, China, and found that the use of hepatotoxic drugs was the most important risk factor for liver injury. In clinical practice, the prevention of drug-induced liver injury is also a key concern in the treatment of COVID-19. Using the updated Roussel Uclaf Causality Assessment Method (RUCAM) can help us determine a causal relationship between drug use and liver injury.^[56]^ The final score corresponds to the following causality levels: ≤0 points, excluding causality; 1–2, unlikely; 3–5, possibly; 6–8 points, possible; and ≥9, very likely.^[56]^ Antiviral drugs used to treat COVID-19, such as remdesivir, lopinavir-ritonavir, and albidol, are considered potentially hepatotoxic and require high level of concern in treatment.^[68]^ In 2020, Muhović et al^[69]^ first reported a case of COVID-19 with severe drug-induced liver injury secondary to tocilizumab, and the RUCAM assessed tocilizumab on drug-induced liver injury with a score of 8. Kumar et al^[70]^ also reported that 3 COVID-19 patients who developed acute cholestatic jaundice after taking favipiravir with RUCAM scores of 7. A study using the updated RUCAM to evaluate 160 patients with COVID-19 found that the most common hepatotoxic drugs used in COVID-19 treatment were hydroxychloroquine, azithromycin, tocilizumab, and ceftriaxone, and the most common type of liver injury secondary to it was hepatocellular.^[71]^ In addition, studies have also found that dexamethasone can increase liver lipid peroxidation, reduce antioxidant activity, and induce elevated liver enzymes in the treatment of COVID-19 patients.^[72]^

This study found that the use of antibiotics and antivirals was not associated with the development of liver injury, while the use of hormones, hydroxychloroquine, and tocilizumab may increase the risk of liver injury. Due to the small literature reporting on drug therapy in this meta-analysis, and all included retrospective analyses, insufficient data were obtained for causal assessment, and additional sample sizes are needed for further analysis in the future. Clinically, it is also recommended that medical staff carefully judge whether the drugs used are hepatotoxic and be vigilant against the occurrence of drug-induced liver injury. In the treatment of COVID-19 patients, we often need to combine the application of multiple drugs, which means that the risk of drug-induced liver injury is greatly increased, and the timely use of the updated RUCAM to assess the risk of drug-induced liver injury can help us identify drug-induced liver injury early.^[73]^

Patients with COVID-19 infection may present asymptomatic or symptomatic, and mild cases usually present with fever, cough, and fatigue. Once severe disease progresses, patients may develop acute respiratory failure, sepsis, multi-organ failure, and even death. This meta-analysis found that patients with COVID-19 who had liver injury were more likely to progress to severe cases, had a longer hospital stay, and had a higher risk of death than patients without liver injury, so early evaluation and intervention are important.

Compared with previous meta-analyses, this study synthesizes a large number of literature, expands the sample size, and performs statistical analysis on more indicators, which is a supplement to previous studies, and the conclusions reached are real and reliable, which is of guiding significance to clinical practice. Our study also had some limitations. First, the studies failed to match potential confounding factors, such as drinking history, hypertension, and diabetes, so there was greater heterogeneity in some data comparisons. Second, the included studies were all published and negative results or small sample sizes were not easy to publish, so we could not rule out potential publication bias.

In summary, this meta-analysis found that the incidence of liver injury in COVID-19 patients was relatively high, affected by age, gender, chronic liver disease, inflammatory state in the body and medication, and COVID-19 patients with liver injury were hospitalized longer and were more likely to have a poor prognosis. The adverse prognostic factors of COVID-19 patients should be identified early in clinical practice, and early intervention should be carried out. For example, we should pay more attention to changes in liver function in middle-aged and elderly men. For patients with prior liver disease, the choice of drugs to treat COVID-19 should take into account their immunocompromised status and underlying liver disease status, and carefully select drugs to avoid secondary liver injury. For the treatment of patients with COVID-19, the RUCAM score is recommended to assess the risk of drug-induced liver injury when a combination of drugs is required. At the same time, patients with severe COVID-19 should be monitored more intensively or individualized to reduce the occurrence of liver injury and improve patient outcomes.

## Author contributions

**Conceptualization:** Xinghai Li.

**Data curation:** Xinghai Li.

**Formal analysis:** Xinghai Li.

**Funding acquisition:** Ning Zhang.

**Investigation:** Xinghai Li.

**Methodology:** Xinghai Li.

**Project administration:** Xinghai Li.

**Resources:** Ning Zhang, Jin Tang.

**Software:** Ning Zhang.

**Supervision:** Xinghai Li, Caiping Fan.

**Validation:** Caiping Fan, Jin Tang.

**Visualization:** Caiping Fan, Ning Zhang, Jin Tang.

**Writing – review & editing:** Jin Tang.
